# Staying safe in the community: adaptation of WaySafe to help probationers make better decisions about their health risks

**DOI:** 10.1186/1940-0640-10-S1-A32

**Published:** 2015-02-20

**Authors:** Wayne EK Lehman, Jennifer Pankow, Kevin Knight, Grace A Rowan, Julie Gray

**Affiliations:** 1 Institute of Behavioral Research, Texas Christian University, Fort Worth, TX, 76129, USA

## 

Transition from incarceration to the community is a critical time for offenders, especially those with substance abuse problems. Many succumb to temptation to return to drug use and possibly risky needle sharing, and engaging in risky sex activities. This critical time period entails substantial health risks for the offender as well as significant risks to public health.

Well-established and consistent use of HIV/HBV/HCV risk reduction prevention programs with continuity of care do not exist in most criminal justice treatment systems because of lack of policy development and integration between institution and community-based corrections, health, and social service agencies. Interventions targeting re-entry are crucial because of the likelihood for risk behaviors to increase upon return to the community. Approaches for community correction populations are needed that have the capability of addressing motivational, social, and cognitive deficits.

This presentation discusses an approach to translating ideas from the prison-based *WaySafe* curriculum into community settings for probationers who have recently completed residential, intensive outpatient, or prison-based substance abuse treatment. The adapted intervention, *StaySafe*, will be based on evidence-based cognitive principles, including TCU Mapping Enhanced Counseling techniques designed to improve decision-making skills regarding health risk behaviors during the critical first 6 months under community supervision. It is being designed to be self-administered by participating probationers during downtime, either prior to or following meetings with their probation officers.

An important goal in the adaptation and testing of *StaySafe* is to have a sustainable, evidence-based product for probation departments that can be administered with minimal staff training and time that is engaging and easy to use by probationers, requires minimal maintenance, and is free to probation departments (other than the cost of the touch-screen computers).

In this presentation, we describe the *StaySafe* intervention and the adaptation process from a prison-based group curriculum to a community corrections-based individualized approach. The adaptation process borrows ideas from the ADAPT-ITT framework [1]. We will describe initial results from focus groups and interviews with probationers and probation officers on problems and successes in probation, logistical issues with delivering interventions in probation settings, and results of a “theater” test of elements of *StaySafe* to gauge participant reactions.
